# Advancing NSCLC pathological subtype prediction with interpretable machine learning: a comprehensive radiomics-based approach

**DOI:** 10.3389/fmed.2024.1413990

**Published:** 2024-05-22

**Authors:** Bingling Kuang, Jingxuan Zhang, Mingqi Zhang, Haoming Xia, Guangliang Qiang, Jiangyu Zhang

**Affiliations:** ^1^Department of Pathology, Affiliated Cancer Hospital and Institution of Guangzhou Medical University, Guangzhou, China; ^2^Nanshan College, Guangzhou Medical University, Guangzhou, Guangdong, China; ^3^The Second Clinical School of Guangzhou Medical University, Guangzhou Medical University, Guangzhou, Guangdong, China; ^4^School of Clinical Medicine, Tsinghua University, Beijing, China; ^5^Department of Thoracic Surgery, Peking University Third Hospital, Beijing, China

**Keywords:** histological subtype, non-small cell lung cancer, interpretable machine learning, CT, radiomics

## Abstract

**Objective:**

This research aims to develop and assess the performance of interpretable machine learning models for diagnosing three histological subtypes of non-small cell lung cancer (NSCLC) utilizing CT imaging data.

**Methods:**

A retrospective cohort of 317 patients diagnosed with NSCLC was included in the study. These individuals were randomly segregated into two groups: a training set comprising 222 patients and a validation set with 95 patients, adhering to a 7:3 ratio. A comprehensive extraction yielded 1,834 radiomic features. For feature selection, statistical methodologies such as the Mann–Whitney U test, Spearman’s rank correlation, and one-way logistic regression were employed. To address data imbalance, the Synthetic Minority Over-sampling Technique (SMOTE) was utilized. The study designed three distinct models to predict adenocarcinoma (ADC), squamous cell carcinoma (SCC), and large cell carcinoma (LCC). Six different classifiers, namely Logistic Regression, Support Vector Machine, Decision Tree, Random Forest, eXtreme Gradient Boosting (XGB), and LightGBM, were deployed for model training. Model performance was gauged through accuracy metrics and the area under the receiver operating characteristic (ROC) curves (AUC). To interpret the diagnostic process, the Shapley Additive Explanations (SHAP) approach was applied.

**Results:**

For the ADC, SCC, and LCC groups, 9, 12, and 8 key radiomic features were selected, respectively. In terms of model performance, the XGB model demonstrated superior performance in predicting SCC and LCC, with AUC values of 0.789 and 0.848, respectively. For ADC prediction, the Random Forest model excelled, showcasing an AUC of 0.748.

**Conclusion:**

The constructed machine learning models, leveraging CT imaging, exhibited robust predictive capabilities for SCC, LCC, and ADC subtypes of NSCLC. These interpretable models serve as substantial support for clinical decision-making processes.

## 1 Introduction

Lung cancer ranks among the top causes of cancer-related deaths globally (1). The two major types of lung cancer are small cell lung cancer (SCLC) and non-small cell lung cancer (NSCLC). The WHO divides NSCLC, which affects 85% of patients, into three primary categories: adenocarcinoma (40%), squamous cell carcinoma (25–30%), and large cell carcinoma (5–10%) (2, 3). Treatments and prognoses vary for NSCLC depending on the histological subtypes (4). For example, Li et al. (5) found that in comparison to non-SQ-NSCLC, ICI monotherapy for SQ-NSCLC led to a noticeably greater survival rate. In addition, Baine et al. (6) showed that SCC is independently linked to an increased risk of death, and patients with SCC who have received SBRT treatment are at an elevated risk of both local and distant failure. In summary, early and accurate diagnosis of NSCLC histological subtypes is essential for the subsequent specific clinical treatment plans.

Until now, the gold standard to diagnose pathological NSCLC subtypes are still CT-guided biopsy and postoperative pathological tissue sections. These methods do have certain drawbacks, though. First of all, these are intrusive tests that have a risk of several complications, including bleeding, air embolism, and pneumothorax (7). In a population-level retrospective study, the percentage of patients who developed comorbidities within 3 days after transthoracic needle biopsy (TTNB) reached 25.8%, with the top three being pneumothorax at 23.3%, hemorrhage, and air embolism (8). Additionally, patients experiencing complications from TTNB demonstrate a heightened likelihood of developing respiratory failure compared to those without complications. Consistent with prior research findings, individuals encountering complications tend to have prolonged hospital stays on average. Simultaneously, smokers, patients of older age, are more prone to postoperative complications (9). Besides, they can end up costing the patients more money and time (10).

Compared to invasive and complex biopsies, a more convenient test is needed to help clinicians make the initial determination of pathological subtypes in patients with NSCLC. The term “radiomics” refers to the automated or semi-automatic post-processing techniques used to analyze various features extracted from imaging exams, and reveals the correlation between these quantitative features and clinical histology or biomarkers (11). In recent years, numerous research have validated the great potential of machine learning combined with radiomics for accurate recognition of histological subtypes, molecular subtypes, and clinical outcome prediction (12–14). Similarly, radiomics has demonstrated comparable success in identifying lung cancer pathological subtypes in previous studies (15, 16). The great majority of previous studies, however, that predict the pathological subtypes of NSCLC have concentrated on differentiating between the two pathological subtypes of ADC and SCC or the three subtypes of ADC, SCC, and SCLC. Few research have been conducted in the recognition of ADC, SCC, and LCC, and existing studies are still deficient in predicting more precise NSCLC pathological subtypes. As medical imaging technology advances, it is now possible to do radiomics analysis on CT, MRI, and PET data. The radiomics workflow remains mostly consistent across these imaging modalities (17). Yet, for the respiratory system, especially in the field of lung cancer, CT is the most common imaging modality. With less radiation exposure than PET/CT and less time and money spent on CT than MRI, CT still has an irreplaceable place in imaging.

Machine learning (ML) has the potential to greatly enhance the accuracy and efficiency of diagnosis across a broad spectrum of diseases, owing to its capacity for processing extensive datasets. ML algorithms are instrumental in refining radiology diagnostic procedures. Through the analysis of medical imagery and supplementary data, models that are adept at identifying disease markers and patterns empower clinical doctors to achieve greater diagnostic accuracy (18). Even so, several prior studies that employed machine learning for the differentiation of NSCLC histological subtypes failed to tackle the challenge of data imbalance. This oversight potentially skewed the outcomes of the final models in favor of categories represented by a larger volume of data, which might affect the accuracy (19, 20). In addition, previous studies have not focused on the interpretability of radiomics, which is not conducive to opening the “black box” of machine learning.

This study aims to build machine learning models based on CT images to noninvasively and accurately predict NSCLC histological subtypes (ADC, SCC, and LCC). In doing so, we addressed the data imbalance issue, which is a common challenge in medical imaging analysis, ensuring a more reliable and robust model performance. Furthermore, we enhanced the transparency and understandability of our models by employing the SHAP (Shapley Additive Explanations) method. This approach not only provided a detailed interpretation of the model’s predictive behavior but also illuminated the significance of individual features in the decision-making process, thereby contributing to a more informed and trustworthy clinical decision-making framework.

## 2 Materials and methods

### 2.1 Patients

Conducted retrospectively, this study utilized The Cancer Imaging Archive (TCIA) database, supported by funding from the Cancer Imaging Program (CIP). The TCIA houses an extensive collection of medical images depicting cancer, which we obtained from the LUNG1 (21) in NSCLC-Radiomics [NSCLC-RADIOMICS–The Cancer Imaging Archive (TCIA)]. Leveraging the publicly accessible resources of The Cancer Imaging Archive (TCIA), this study did not necessitate ethical approval. From the MAASTRO Clinic in the Netherlands, a cohort of 422 patients diagnosed with NSCLC was selected. Comprehensive data, including clinical outcomes, survival statistics, CT imaging, and histological subtypes, were accessible for these patients. Histological classifications were determined using surgical specimens. Exclusions were made for 42 patients lacking pathological confirmation and 63 patients classified as “not otherwise specified” (NOS), resulting in 317 patients being eligible for inclusion in the model-building process. Of these, 51 were diagnosed with ADC, 152 with SCC, and 114 with LCC.

### 2.2 Region of interest interception

In our study, the DICOM Radiation Therapy Structure Set (RTSTRUCT) and DICOM Segmentation (SEG) files were integral for delineating the regions of interest (ROI) within the CT images. These files encapsulate the manual segmentation efforts conducted by experienced radiation oncologists, who meticulously outlined the ROIs pertinent to the NSCLC histological subtypes.

To ensure the utmost accuracy and reliability of the segmentation, we undertook a comprehensive verification process for each annotated ROI. This involved a rigorous review by 2 physicians with more than 3 years of experience in radiology (B. Kuang and M. Zhang), who cross-examined the segmented areas against the corresponding histological findings. The process was iterative, with each ROI being scrutinized for precision in demarcating the tumor boundaries and its conformity to the recognized pathological characteristics of the NSCLC subtypes.

### 2.3 Radiomic features

#### 2.3.1 Radiomic features extraction

Features extraction was based on Python 3.7 and implemented using the PyRadiomics software.^1^ Radiomic features extracted from CT images are categorized into geometric, intensity, and texture features, each capturing different aspects of the tumor’s characteristics within the ROI. Geometric features delineate the 3D shape of the ROI, detailing aspects like the volume, surface area, and the overall spatial configuration of the tumor. They help in understanding the physical dimensions and shape irregularities of the tumor mass. Intensity features, on the other hand, deal with the first-order statistical distribution of voxel intensities inside the ROI, providing insights into the density and uniformity of the tumor tissue through metrics such as mean intensity, standard deviation, skewness, and kurtosis.

Texture features go a step further by describing the second-order and higher-order spatial distribution of voxel intensities, reflecting the heterogeneity within the tumor. These features are extracted using methods like the gray level co-occurrence matrix (GLCM), which evaluates how often pairs of pixel with specific values and in a specified spatial relationship occur in an image, or the gray level run length matrix (GLRLM), which considers the length of contiguous runs of pixels having the same gray level value. Other methods include the gray level size zone matrix (GLSZM), which assesses the distribution of different-sized zones of similar gray level values, and the neighborhood gray-tone difference matrix (NGTDM), which quantifies the difference in gray-level values between a pixel and its surrounding neighbors. Together, these texture features provide a comprehensive view of the textural patterns and complexity of the tumor, offering crucial insights into its pathological and physiological state.

#### 2.3.2 Radiomic features selection

In the process of radiomic features selection, we first subjected all imaging features to the Mann–Whitney U statistical test to identify significant differences between groups. Only features that demonstrated a statistically significant difference, with a *p*-value less than 0.05, were retained for further analysis. Following this initial filtration, we applied the Spearman rank correlation coefficient to evaluate the interrelationships among the features. This step was crucial to identify and eliminate highly correlated features, with a threshold set at a coefficient value above 0.9, to reduce redundancy and potential collinearity in the dataset. To refine the feature set further, we conducted one-way logistic regression on the remaining features, again selecting only those with a *p*-value less than 0.05. This method ensured that the final set of features had both statistical significance and predictive relevance.

### 2.4 Machine learning models

In this study, we developed three distinct machine learning models tailored to the pathological subtypes of NSCLC: ADC, SCC, and LCC. Each model was specifically constructed to predict the likelihood of one of these subtypes based on the radiomic features extracted from the CT images.

To ensure the robustness and accuracy of our predictions, we employed six different classifiers for training each model. These classifiers included Logistic Regression (LR), Support Vector Machine (SVM), Decision Tree (DT), Random Forest (RF), eXtreme Gradient Boosting (XGB), and LightGBM (LGBM). Each of these classifiers brings unique strengths and approaches to the modeling process, such as LR’s ability to provide linear decision boundaries, SVM’s effectiveness in high-dimensional spaces, DT’s clear decision rules, RF’s ensemble learning for reducing overfitting, XGB’s optimization in gradient boosting, and LGBM’s efficiency in handling large data sets. The training process included a 5-fold cross-validation on the training set to optimize the model parameters. Following this, an independent test on the validation set was conducted to evaluate the models’ performance, ensuring that our predictive models were both robust and generalizable. The average area under the curve (AUC) was utilized to assess the accuracy of predictive models.

### 2.5 Stata analysis

For statistical analysis in our experiment, we employed the R software version 4.2.3. The Shapiro-Wilk test was utilized to evaluate the normality of the measurement data, which were expressed as mean ± standard deviation (SD) for normally distributed data. Non-normally distributed data were presented as medians with first and third quartiles (Q1, Q3), and categorical data were expressed as counts and percentages (*n*, %). For comparing measured data, we used the independent samples *t*-test for normally distributed data and the Mann–Whitney U-test for non-normally distributed data. The chi-squared (χ^2^) test was applied for comparing count data. Statistical significance was established at a *p*-value of less than 0.05. The predictive efficacy of the models was assessed using receiver operating characteristic (ROC) curves and accuracy measurements.

The entire dataset of 317 samples was randomly divided into training and validation sets in a 7:3 ratio. To mitigate the challenge of unbalanced data, we implemented the Synthetic Minority Over-sampling Technique (SMOTE). This method is crucial for augmenting the minority class in the dataset by synthesizing new samples, thereby achieving a balance in the class distribution. This balanced dataset was then used to enhance the performance of our classification models (22). The prediction model with the highest AUC was selected as the optimal choice. ROC curves were utilized to evaluate the predictive performance of various models in forecasting NSCLC pathological subtypes. In addition, we also visualized the results of the model through SHAP analysis. The importance of each radiomic feature is ranked.

## 3 Results

### 3.1 Patient baseline characteristics

In our study, we included 317 patients diagnosed with non-small cell lung cancer (NSCLC), featuring a mean age of 68.5 years. The distribution of histological subtypes among these patients was as follows: 51 were diagnosed with ADC, 152 with SCC, and 114 with LCC. The baseline characteristics of these patients, including demographic details, clinical stage, and other relevant clinical parameters, are meticulously cataloged in Table 1. This comprehensive data set provides a foundational understanding of the patient demographics and disease specifics, serving as a critical reference point for the predictive efficacy of the developed machine learning models.

**TABLE 1:** The patient’s clinical baseline data and statistical results.

	Test	Train	*P*-value
	*N* = 96	*N* = 221	
Age	67.0 [62.0;78.0]	70.0 [62.0;76.0]	0.919
T_stage:			0.702
1	18 (18.8%)	37 (16.7%)	
2	36 (37.5%)	88 (39.8%)	
3	13 (13.5%)	30 (13.6%)	
4	28 (29.2%)	66 (29.9%)	
5	1 (1.04%)	0 (0.00%)	
N_stage:			0.343
0	35 (36.5%)	78 (35.3%)	
1	3 (3.12%)	18 (8.14%)	
2	34 (35.4%)	79 (35.7%)	
3	24 (25.0%)	43 (19.5%)	
4	0 (0.00%)	3 (1.36%)	
M_stage:			0.027
0	93 (96.9%)	221 (100%)	
3	3 (3.12%)	0 (0.00%)	
Stage:			0.411
I	19 (20.0%)	30 (13.6%)	
II	8 (8.42%)	28 (12.7%)	
IIIa	28 (29.5%)	66 (29.9%)	
IIIb	40 (42.1%)	97 (43.9%)	
Histology:			0.757
Adenocarcinoma	15 (15.6%)	36 (16.3%)	
Large cell	32 (33.3%)	82 (37.1%)	
Squamous cell carcinoma	49 (51.0%)	103 (46.6%)	
Gender:			0.532
Female	28 (29.2%)	74 (33.5%)	
Male	68 (70.8%)	147 (66.5%)	

### 3.2 Radiomic feature extraction and selection results

The radiomic analysis of the CT images from the 317 NSCLC patients resulted in the extraction of 1,834 distinct features. These features encompassed a broad spectrum of geometric, intensity, and texture characteristics, providing a comprehensive dataset for subsequent analysis.

**FIGURE 1:**
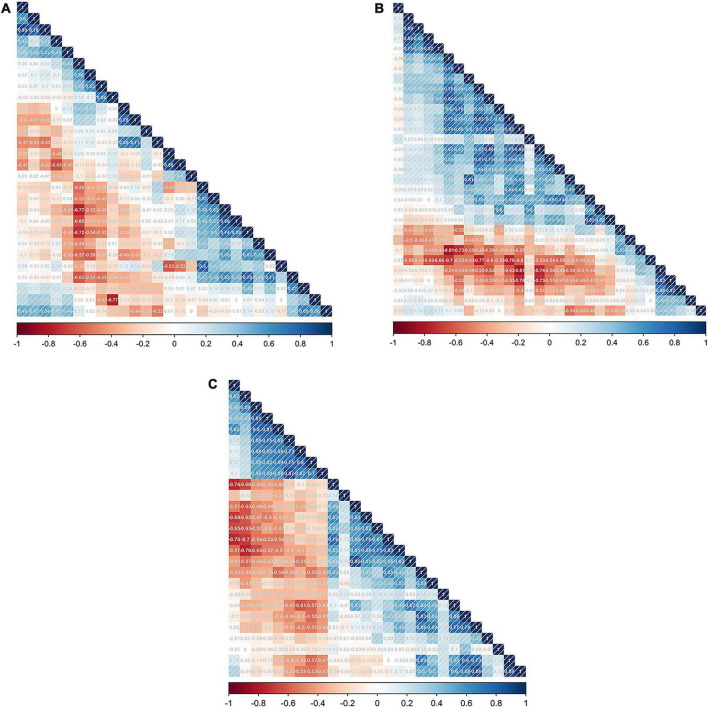
The feature correlation matrix obtained by performing Spearman’s rank correlation analysis in the SCC **(A)**, LCC **(B)**, and ADC groups **(C)**, respectively.

To refine this extensive set of features and identify the most predictive ones for each NSCLC subtype, we applied a series of statistical methods. Initially, the Mann–Whitney U test was used to filter out features that showed significant differences between the subtypes, ensuring that the retained features had potential diagnostic value. This was followed by the Spearman’s rank correlation analysis, which helped in identifying and eliminating features that were highly correlated with others, thereby reducing redundancy and focusing on those features that offered unique information. For the final stage of feature selection, one-way logistic regression was performed, further narrowing down the feature set to those with significant predictive power, as indicated by *p*-values less than 0.05. As a result, the feature selection process culminated in the identification of 9 key radiomic features for the ADC group, 12 for the SCC group, and 8 for the LCC group, which was shown in Table 2. These selected features represent the most relevant and informative characteristics for predicting the histological subtypes of NSCLC. To visually represent the inter-relationships and correlations among the selected features, Spearman correlation heatmaps were created for each group. These heatmaps (Figures 1A–C) provide a graphical illustration of the feature correlations, with varying intensities of color indicating the strength and direction of the correlations.

**TABLE 2:** The key radiomic features for the SCC, ADC and LCC group.

	SCC	ADC	LCC
Key features	lbp_3D_m1_glcm_Idmn	lbp_3D_k_glszm_GrayLevelVariance	log_sigma_1_0_mm_3D_ngtdm_Complexity
	lbp_3D_m1_glszm_GrayLevelVariance	lbp_3D_m1_glszm_SmallAreaLow GrayLevelEmphasis	log_sigma_2_0_mm_3D_firstorder_90Percentile
	lbp_3D_m1_glszm_LowGrayLevel ZoneEmphasis	log_sigma_2_0_mm_3D_ngtdm_Busyness	log_sigma_3_0_mm_3D_firstorder_Skewness
	lbp_3D_m2_glszm_HighGrayLevel ZoneEmphasis	wavelet_HHH_ngtdm_Busyness	original_glcm_ClusterShade
	lbp_3D_m2_glszm_LowGrayLevel ZoneEmphasis	wavelet_HHL_firstorder_RootMeanSquared	original_gldm_SmallDependenceHighGray LevelEmphasis
	lbp_3D_m2_glszm_SizeZoneNonUniformity Normalized	wavelet_LHL_firstorder_Maximum	square_firstorder_Entropy
	lbp_3D_m2_glszm_SmallAreaHighGray LevelEmphasis	wavelet_LHL_firstorder_Minimum	square_glcm_Correlation
	log_sigma_1_0_mm_3D_firstorder _90Percentile	wavelet_LHL_firstorder_RootMeanSquared	square_glcm_Idm
	log_sigma_1_0_mm_3D_glcm _Correlation	wavelet_LHL_ngtdm_Busyness	
	log_sigma_3_0_mm_3D_firstorder _Skewness		
	wavelet_HHH_ngtdm_Busyness		
	wavelet_HLH_ngtdm_Busyness		

### 3.3 Prediction model results

#### 3.3.1 Distinguish the SCC subtype from all other subtypes

**FIGURE 2:**
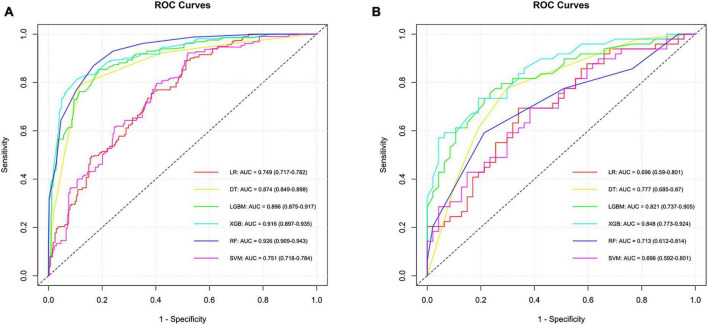
ROC curves and corresponding AUC values in the training **(A)** and testing **(B)** sets of the SCC group.

Predictive modeling for SCC was conducted using six different algorithms, yielding the following AUC values in the cross-validation set: LR exhibited an AUC of 0.749 (95% CI, 0.717–0.782), SVM showed 0.751 (95% CI, 0.718–0.784), RF achieved 0.926 (95% CI, 0.909–0.943), DT had 0.874 (95% CI, 0.849–0.898), XGB reached 0.916 (95% CI, 0.897–0.935), and LGBM recorded 0.896 (95% CI, 0.875–0.917). In the testing set, the AUC values were consistent with those in the training set for LR, SVM, RF, DT, XGB, and LGBM, demonstrating the models’ stability and generalizability. Specifically, the AUC for each algorithm remained as follows: LR at 0.696 (95% CI, 0.590–0.801), SVM at 0.696 (95% CI, 0.592–0.801), RF at 0.713 (95% CI, 0.612–0.814), DT at 0.777 (95% CI, 0.685–0.87), XGB at 0.848 (95% CI, 0.773–0.924), and LGBM at 0.821 (95% CI, 0.737–0.905). Notably, the XGB model outperformed others in differentiating SCC from ADC and LCC within the testing set, evidenced by an AUC of 0.848 (95% CI, 0.773–0.924) and an accuracy rate of 0.750. This indicates the superior predictive capability of the XGB algorithm in the context of this study. The relevant model evaluation index results are shown in Supplementary material 1, and the corresponding ROCs curve are shown in Figure 2.

#### 3.3.2 Distinguish the LCC subtype from all other subtypes

**FIGURE 3:**
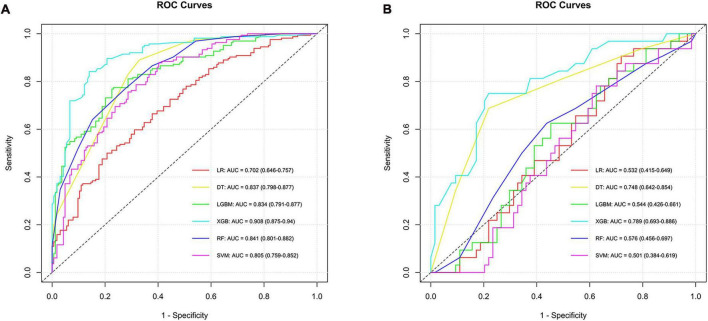
ROC curves and corresponding AUC values in the training **(A)** and testing **(B)** sets of the LCC group.

In the construction of models to predict LCC, six algorithms were employed, resulting in varied AUC values in the training set: LR achieved an AUC of 0.702, SVM attained 0.805, RF reached 0.841, DT had 0.837, XGB stood out with 0.908, and LGBM was at 0.834, with their 95% confidence intervals ranging from 0.646 to 0.940 across the different methods. In the testing phase, the performance of these algorithms showed a different pattern of AUC values, indicating varying levels of efficacy in model generalization. LR presented an AUC of 0.532, SVM at 0.501, RF at 0.576, DT scored 0.748, XGB led with 0.789, and LGBM recorded 0.544, with their 95% confidence intervals demonstrating the consistency in model performance under varying conditions. The XGB model emerged as the most effective in differentiating LCC from ADC and SCC in the testing set, showcasing its superior predictive ability among the tested algorithms. Similarly, the performance indicators and ROC curves of the model are shown in Supplementary material 1 and Figure 3.

#### 3.3.3 Distinguish the ADC subtype from all other subtypes

**FIGURE 4:**
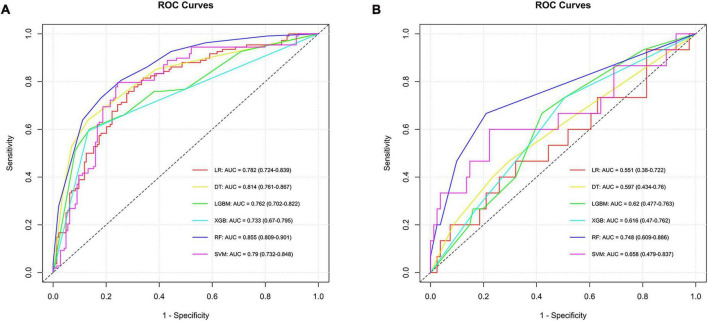
ROC curves and corresponding AUC values in the training **(A)** and testing **(B)** sets of the ADC group.

To identify the ADC subtype, models were developed using six algorithms, yielding AUC values in the training set as follows: LR had 0.782, SVM 0.790, RF 0.855, DT 0.814, XGB 0.733, and LGBM 0.762, with confidence intervals ranging from 0.670 to 0.901. In the testing set, AUC values were LR at 0.551, SVM at 0.658, RF at 0.748, DT at 0.597, XGB at 0.616, and LGBM also at 0.620, indicating varying levels of predictive performance. The RF model stood out for its ability to differentiate ADC, achieving an AUC of 0.748 in the testing set, thus proving to be the most effective among the algorithms tested for this subtype (Supplementary material 1 and Figure 4).

### 3.4 Model explanation

**FIGURE 5:**
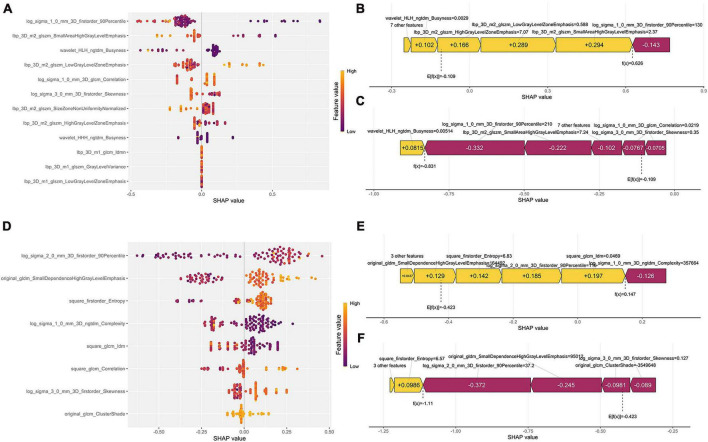
Model interpretability display. **(A,D)** Represent the SHAP plots for the SCC and LCC groups, respectively; **(B,C)** show the radiomics feature force plots for two random patients in the SCC group (predicted as SCC and non-SCC), respectively; **(E,F)** are the radiomics feature force plots for two random patients in the LCC group (predicted as LCC and non-LCC), respectively.

The XGB model with the best prediction in differentiating SCC from the other two subtypes was used to demonstrate the SHAP plots. Figure 5A depicts the ranking of importance of the 12 features. The two most important features were log sigma 1.0 mm 3D firstorder 90Percentile and lbp 3D m2 glszm LowGrayLevelZoneEmphasis. The decision-making process of the XGB model for two patients is depicted using the SHAP force diagram (Figures 5B, C). The score calculation begins with E[f(x)] and then sums the SHAP values, with yellow representing an increased probability or purple representing a decreased probability of squamous carcinoma, ending with the individual prediction. Similarly, the explanatory display of the LCC groups is shown in Figures 5D–F (It is worth noting that since the algorithm mechanism of RF cannot display the same SHAP and force diagram as above, the ADC group is not displayed.).

## 4 Discussion

Accurate identification of histological subtype of patients with NSCLC has important implications for both clinical therapeutic approaches and patient prognosis. In our study, we developed CT image-based ML models for distinguishing different pathological subtypes of NSCLC patients and compared the performance of each ML model. Ultimately, our model can assist clinicians in diagnosis and suggest to them those imaging features that are important in distinguishing NSCLC pathological subtypes.

The pre-selected features screened for model building belong to first order features and texture features so as the two most important features for distinguishing SCC and the other two subtypes in the XGB model, which were similar with the previous studies (20). CT images have good spatial resolution and can represent different tissue structures in grayscale, while the texture of CT images may be related to the heterogeneity of the tumor and may predict the biological behavior of the tumor (23). The XGB model performed best both in differentiating SCC and LCC, reaching an AUC of 0.848 and 0.789, respectively. The RF model outperformed the other five models, discriminating between ADC and non-ADC with an AUC of 0.748. There may be several reasons for this phenomenon. One reason for this is that no algorithm can maintain the best in all datasets (24). The above evidence suggested that our models were reliable.

Nowadays, there are many studies based on imaging to differentiate histological types of lung cancer. Numerous studies have shown that imaging features can be used to identify the histological type of NSCLC patients. Guo et al. (20) developed two models, the ProNet model and com_radNet, based on CT scans. The AUCs were 0.840 and 0.789, with corresponding accuracy of 71.6 and 74.7%. Zhou et al. (25) used nine machine learning classifiers to construct 45 prediction models after extracting radiomic features from PET and CT scans. AUC (0.897) for the GBDT feature selection method combined GBDT classifier was highest in the PET dataset; AUC (0.839) for the GBDT feature selection method and RF classifier was highest in the CT dataset. Furthermore, some studies have combined clinical features, tumor markers, and radiomic features to construct models. Hyun et al. (26) investigated 4 clinical features and 40 imaging features extracted from PET images to build a prediction model by five machine learning algorithms, the logistic regression model, which was the best predictor, with an AUC of 0.859. Zhao et al. (27) evaluated 13 different characteristics for modeling. These included 2 clinical variables (gender and smoking status), 2 laboratory markers (CEA and SCCA), and 9 radiological features. The researchers were able to achieve an AUC value of 0.910 in the evaluation of the test set. Yan and Wang (28) further investigated by establishing the PET model, CT model, and combined PET and CT model, and found that the combined PET and CT model had the best performance in predicting ADC, SCC, and metastasis. Deep learning algorithms also hold promise for predicting NSCLC histological subtypes. Wang et al. (29) discovered that the deep learning model they developed to predict the histological subtypes of ADC performed well on 2-classification, 3-classification, and even 8-classification. They did this by extracting features from CT scans.

Our study built three prediction models for ADC, SCC, and LCC with AUCs of 0.748, 0.848, and 0.789, respectively, and further demonstrated the feature importance ranking of the models as well as the decision-making process. To date, published studies have focused on distinguishing ADC from SCC, or SCLC. A study by Zhao et al. (30) established a Mobilenet v2 model for discriminating ADC and SCC, yielding an AUC value of 0.767, slightly lower than ours. In this study, we innovatively constructed 3 models to predict ADC, SCC, and LCC, respectively, whereas very few prediction models involving LCC subtype have been previously developed. Consequently, our study has contributed to a more refined and precise prediction of pathological subtypes in NSCLC. As well, few studies have dealt with the issue of data imbalance before. The study by Lin et al. (19), which did not deal with data imbalance, built a model with an AUC of only 0.700, significantly lower than our model. Therefore, we provide the possibility for the development of more accurate models in the future.

In addition, due to the black-box nature of machine learning (31), machine learning models lack interpretability in previous studies. Linning et al. (32) extracted features from CT images and built models to distinguish SCLC, ADC and SCC with AUC values of 0.822 and 0.665, respectively, but they failed to further explain the importance ranking of the features and the machine-learning decision-making process. We implemented the SHAP method. This technique illuminated the decision-making process of our models by providing a clear ranking of feature importance and delineating how each feature influences predictions. Such transparency is crucial in a clinical context, as it builds trust and aids clinicians in understanding the basis for model predictions, thus facilitating informed decision-making. By revealing the contributions of individual radiomic features to the classification of NSCLC subtypes, our approach not only enhances the trustworthiness and validation of our models but also deepens the understanding of the link between radiomic characteristics and cancer pathology, paving the way for more tailored and effective treatment approaches. There are also some shortcomings in this study. Compared with the results of previous studies, we did not significantly improve the effectiveness of machine learning models based on CT features in predicting NSCLC histological subtypes. In the future, more advanced algorithms and models such as deep learning and end-to-end modeling could be used to predict NSCLC pathological subtypes.

The limitations of this investigation are chiefly found in the following areas. Initially, the data, sourced from public databases, were characterized by a relatively modest sample size and an insufficiently detailed baseline profile, adversely affecting the model’s predictive capability. Attaining improved efficacy would necessitate support from a more substantial research cohort. Secondly, the data, originating from a single center, employed internal data for the prediction model’s validation, rendering the findings preliminary until corroborated by multicenter prospective studies. Lastly, the study exclusively involved imaging features in model development, omitting clinical details such as age and gender, resulting in a comparatively uniform dataset. This limitation may compromise the model’s generalizability in real-world clinical diagnosis.

## 5 Conclusion

This study successfully developed interpretable machine learning models using CT images to diagnose histological subtypes of NSCLC, with the XGB and RF models showing superior performance. The use of SHAP for interpretability further strengthens the clinical relevance of our models, providing insights into the decision-making process and contributing to more informed and transparent diagnostic pathways. Looking ahead, there is an opportunity to build upon this foundation by creating advanced predictive models that integrate data from multiple centers and encompass multi-omics associations.

## Data availability statement

Publicly available datasets were analyzed in this study. This data can be found here: https://www.cancerimagingarchive.net/collection/nsclc-radiomics/.

## Ethics statement

Ethical approval was not required for the study involving humans in accordance with the local legislation and institutional requirements. Written informed consent to participate in this study was not required from the participants or the participants’ legal guardians/next of kin in accordance with the national legislation and the institutional requirements.

## Author contributions

BK: Conceptualization, Data curation, Methodology, Software, Writing – original draft. JinZ: Investigation, Methodology, Writing – original draft. MZ: Data curation, Writing – original draft. HX: Software, Writing – original draft. GQ: Conceptualization, Funding acquisition, Writing – review & editing. JiaZ: Conceptualization, Funding acquisition, Writing – review & editing.

## Footnotes

1
http://pyradiomics.readthedocs.io/en/latest

## Supplementary material

Supplementary Material The model evaluation index results.

## References

[1] Leiter A Veluswamy RR Wisnivesky JP. The global burden of lung cancer: Current status and future trends. *Nat Rev Clin Oncol.* (2023) 20:624–39. 10.1038/s41571-023-00798-3 37479810

[2] Travis WD Brambilla E Nicholson AG Yatabe Y Austin JHM Beasley MB The 2015 world health organization classification of lung tumors. *J Thorac Oncol.* (2015) 10:1243–60. 10.1097/JTO.0000000000000630 26291008

[3] Nicholson AG Tsao MS Beasley MB Borczuk AC Brambilla E Cooper WA The 2021 who classification of lung tumors: Impact of advances since 2015. *J Thorac Oncol.* (2022) 17:362–87. 10.1016/j.jtho.2021.11.003 34808341

[4] Ettinger DS Wood DE Aisner DL Akerley W Bauman JR Bharat A Non–small cell lung cancer, version 3.2022, NCCN clinical practice guidelines in oncology. *J Natl Compr Cancer Netw.* (2022) 20:497–530. 10.6004/jnccn.2022.0025 35545176

[5] Li F Zhai S Lv Z Yuan L Wang S Jin D Effect of histology on the efficacy of immune checkpoint inhibitors in advanced non-small cell lung cancer: A systematic review and meta-analysis. *Front Oncol.* (2022) 12:968517. 10.3389/fonc.2022.968517 36439448 PMC9685340

[6] Baine MJ Verma V Schonewolf CA Lin C Simone CB. Histology significantly affects recurrence and survival following SBRT for early stage non-small cell lung cancer. *Lung Cancer.* (2018) 118:20–6. 10.1016/j.lungcan.2018.01.021 29571997

[7] Dubé J-P Azzi Z Semionov A Sayegh K Kosiuk J Pressacco J. Imaging of post transthoracic needle biopsy complications. *Can Assoc Radiol J.* (2019) 70:156–63. 10.1016/j.carj.2018.08.006 30635216

[8] Vachani A Zhou M Ghosh S Zhang S Szapary P Gaurav D Complications after transthoracic needle biopsy of pulmonary nodules: A population-level retrospective cohort analysis. *J Am Coll Radiol.* (2022) 19:1121–9. 10.1016/j.jacr.2022.04.010 35738412

[9] Wiener RS Schwartz LM Woloshin S Welch HG. Population-based risk of complications following transthoracic needle lung biopsy of a pulmonary nodule. *Ann Intern Med.* (2011) 155:137–44. 21810706 10.1059/0003-4819-155-3-201108020-00003 PMC3150964

[10] Godfrey CM Shipe ME Welty VF Maiga AW Aldrich MC Montgomery C The thoracic research evaluation and treatment 2.0 model. *Chest.* (2023) 164:1305–14. 10.1016/j.chest.2023.06.009 37421973 PMC10635839

[11] Rossi G Barabino E Fedeli A Ficarra G Coco S Russo A Radiomic detection of EGFR mutations in Nsclc. *Cancer Res.* (2021) 81:724–31. 10.1158/0008-5472.CAN-20-0999 33148663

[12] Wang W Liang H Zhang Z Xu C Wei D Li W Comparing three-dimensional and two-dimensional deep-learning, radiomics, and fusion models for predicting occult lymph node metastasis in laryngeal squamous cell carcinoma based on CT imaging: A multicentre, retrospective, diagnostic study. *eClinicalMedicine.* (2024) 67:102385. 10.1016/j.eclinm.2023.102385 38261897 PMC10796944

[13] Ma M Liu R Wen C Xu W Xu Z Wang S Predicting the molecular subtype of breast cancer and identifying interpretable imaging features using machine learning algorithms. *Eur Radiol.* (2022) 32:1652–62. 10.1007/s00330-021-08271-4 34647174

[14] Stüber AT Coors S Schachtner B Weber T Rügamer D Bender A A comprehensive machine learning benchmark study for radiomics-based survival analysis of CT imaging data in patients with hepatic metastases of CRC. *Invest Radiol.* (2023) 58:874–81. 10.1097/RLI.0000000000001009 37504498 PMC10662603

[15] Koyasu S Nishio M Isoda H Nakamoto Y Togashi K. Usefulness of gradient tree boosting for predicting histological subtype and Egfr mutation status of non-small cell lung cancer on 18f Fdg-Pet/CT. *Ann Nucl Med.* (2020) 34:49–57. 10.1007/s12149-019-01414-0 31659591

[16] Bianconi F Palumbo I Fravolini ML Chiari R Minestrini M Brunese L Texture analysis on [18f]Fdg Pet/CT in non-small-cell lung cancer: Correlations between pet features, CT features, and histological types. *Mol Imaging Biol.* (2019) 21:1200–9. 10.1007/s11307-019-01336-3 30847822

[17] Tagliafico AS Piana M Schenone D Lai R Massone AM Houssami N. Overview of radiomics in breast cancer diagnosis and prognostication. *Breast.* (2020) 49:74–80. 10.1016/j.breast.2019.10.018 31739125 PMC7375670

[18] Choy G Khalilzadeh O Michalski M Do S Samir AE Pianykh OS Current applications and future impact of machine learning in radiology. *Radiology.* (2018) 288:318–28. 10.1148/radiol.2018171820 29944078 PMC6542626

[19] Lin J Yu Y Zhang X Wang Z Li S. Classification of histological types and stages in non-small cell lung cancer using radiomic features based on CT images. *J Digit Imaging.* (2023) 36:1029–37. 10.1007/s10278-023-00792-2 36828962 PMC10287608

[20] Guo Y Song Q Jiang M Guo Y Xu P Zhang Y Histological subtypes classification of lung cancers on CT images using 3d deep learning and radiomics. *Acad Radiol.* (2021) 28:e258–66. 10.1016/j.acra.2020.06.010 32622740

[21] Aerts HJWL Velazquez ER Leijenaar RTH Parmar C Grossmann P Carvalho S Decoding tumour phenotype by noninvasive imaging using a quantitative radiomics approach. *Nat Commun.* (2014) 5:4006. 10.1038/ncomms5006 24892406 PMC4059926

[22] Chawla NV Bowyer KW Hall LO Kegelmeyer WP. Smote: Synthetic minority over-sampling technique. *J Artif Intell Res.* (2002) 16:321–57. 10.1613/jair.953

[23] Nishio M Nagashima C. Computer-aided diagnosis for lung cancer. *Acad Radiol.* (2017) 24:328–36. 10.1016/j.acra.2016.11.007 28110797

[24] Zhang Y Xin Y Li Q Ma J Li S Lv X Empirical study of seven data mining algorithms on different characteristics of datasets for biomedical classification applications. *BioMed Eng OnLine.* (2017) 16:125. 10.1186/s12938-017-0416-x 29096638 PMC5668968

[25] Zhou Y Ma X-L Zhang T Wang J Zhang T Tian R. Use of radiomics based on 18f-Fdg Pet/CT and machine learning methods to aid clinical decision-making in the classification of solitary pulmonary lesions: An innovative approach. *Eur J Nucl Med Mol Imaging.* (2021) 48:2904–13. 10.1007/s00259-021-05220-7 33547553

[26] Hyun SH Ahn MS Koh YW Lee SJ. A machine-learning approach using pet-based radiomics to predict the histological subtypes of lung cancer. *Clin Nucl Med.* (2019) 44:956–60. 10.1097/RLU.0000000000002810 31689276

[27] Zhao H Su Y Wang M Lyu Z Xu P Jiao Y The machine learning model for distinguishing pathological subtypes of non-small cell lung cancer. *Front Oncol.* (2022) 12:875761. 10.3389/fonc.2022.875761 35692759 PMC9177952

[28] Yan M Wang W. Development of a radiomics prediction model for histological type diagnosis in solitary pulmonary nodules: The combination of CT and Fdg Pet. *Front Oncol.* (2020) 10:555514. 10.3389/fonc.2020.555514 33042839 PMC7523028

[29] Wang C Shao J Lv J Cao Y Zhu C Li J Deep learning for predicting subtype classification and survival of lung adenocarcinoma on computed tomography. *Transl Oncol.* (2021) 14:101141. 10.1016/j.tranon.2021.101141 34087705 PMC8184655

[30] Zhao H Su Y Lyu Z Tian L Xu P Lin L Non-invasively discriminating the pathological subtypes of non-small cell lung cancer with pretreatment 18f-Fdg Pet/CT using deep learning. *Acad Radiol.* (2024) 31:35–45. 10.1016/j.acra.2023.03.032 37117141

[31] The Lancet Respiratory Medicine. Opening the black box of machine learning. *Lancet Respir Med.* (2018) 6:801. 10.1016/S2213-2600(18)30425-9 30343029

[32] Linning E Lu L Li L Yang H Schwartz LH Zhao B. Radiomics for classification of lung cancer histological subtypes based on nonenhanced computed tomography. *Acad Radiol.* (2019) 26:1245–52. 10.1016/j.acra.2018.10.013 30502076

